# Supplementary material for: Pro-Vegetarian Food Patterns and Cancer Risk among Italians from the Moli-Sani Study Cohort
Source: Nutrients. 2023 Sep 14;15(18):3976. doi: 10.3390/nu15183976 (PMC10535500; doi:10.3390/nu15183976)
Supplement: Supplementary file 1 [file nutrients-15-03976-s001.zip › nutrients-2563611-supplementary.pdf]

Pro-vegetarian food patterns and cancer risk among Italians from the Moli-sani Study cohort

Martínez CF et al.

On-line Supplementary Material

Supplementary Figure S1. Flowchart for selection of study participants from the Moli-sani Study, 2005-2010.

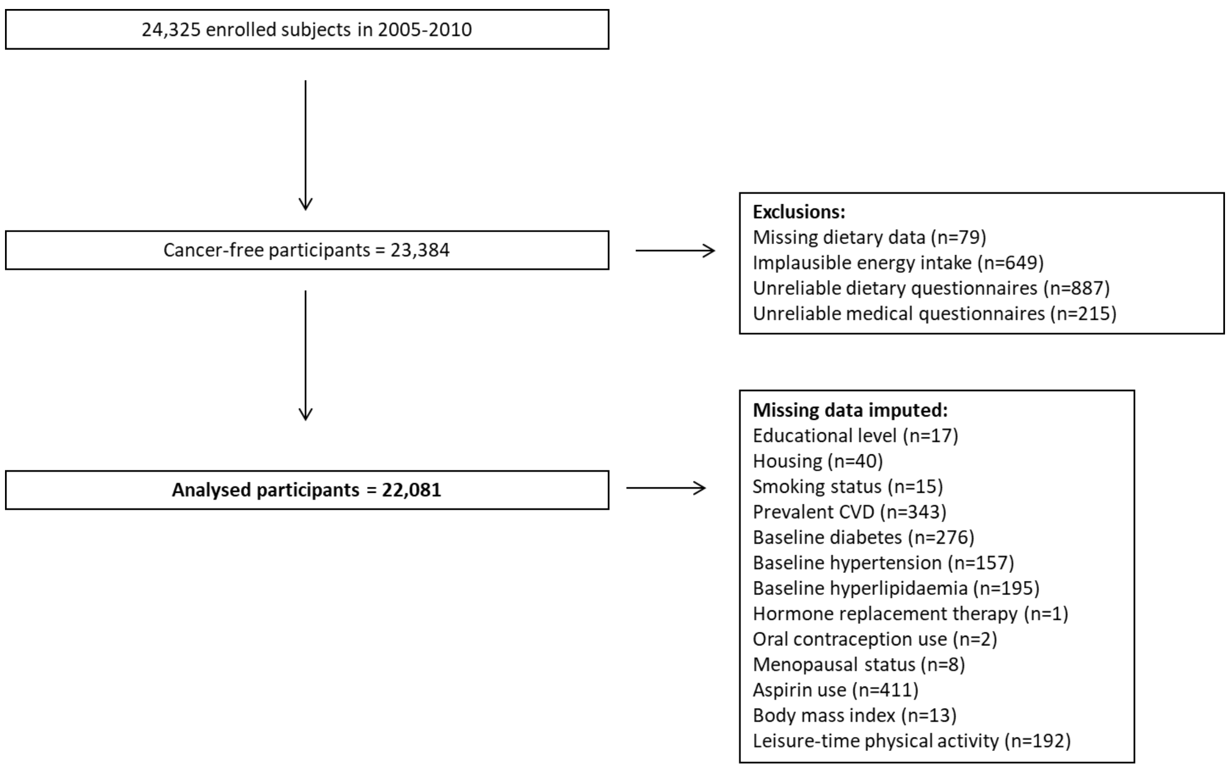

Supplementary Table S1. Scoring criteria for the pro-vegetarian (PVG) food patterns in the Moli-sani Study cohort, Italy 2005-2010.

Component	Included foods	General PVG	Healthful PVG	Unhealthful PVG
Plant food groups (n=12)				
Vegetables	Spinach, turnip greens, salad, green pepper, pumpkin, tomatoes, carrot, beet, broccoli, Brussel sprouts, cauliflower, cabbage, kale, mushrooms, garlic, onions, zucchini, artichoke, fennel, olives	Positive	Positive	Reverse
Fruits	Citrus, apple, pear, banana, kiwi, grape, peach, apricot, prune, strawberries, melon, fruit salad, figs, cherries, persimmon	Positive	Positive	Reverse
Legumes	Beans, chickpeas, lentils, peas, broad beans	Positive	Positive	Reverse
Whole grain	Whole-grain bread	Positive	Positive	Reverse
Refined grains	Crispbread/rusks, breakfast cereals, white bread, other bread, rice, pasta and other grains	Positive	Reverse	Positive
Potatoes	Potatoes	Positive	Reverse	Positive
Nuts and dried fruit	Walnut, hazelnut, almond, peanut, dried fruit	Positive	Positive	Reverse
Olive oil	Common olive oil	Positive	Positive	Reverse
Tea and coffee	Tea, caffeinated coffee, decaffeinated coffee	Not scored	Positive	Reverse
Fruit juices	Fruit juices	Not scored	Reverse	Positive
Sugar-sweetened beverages	Carbonated/soft/isotonic drinks, diluted syrups	Not scored	Reverse	Positive
Sweets and desserts	Chocolate, nut spread, candies, cakes, pies, pastries, puddings (non-milk based), biscuits, dry cakes, honey, jam and sugar	Not scored	Reverse	Positive
Animal food groups (n=5)				
Meat and meat products	Chicken or turkey, rabbit, beef, pork, lamb, veal, offal, ham, cured meats, salami, mortadella, sausage, hamburger	Reverse	Reverse	Reverse
Animal fats for cooking or as a spread	Butter, another animal fat	Reverse	Reverse	Reverse
Eggs	Eggs	Reverse	Reverse	Reverse
Fish and other seafood	Hake, sole, sardines, trout, swordfish, shrimp, prawns, squid, cuttlefish, octopus, clams, stock fish, canned fish	Reverse	Reverse	Reverse
Milk and dairy products	Whole milk, partially skimmed or skimmed milk, plain yogurt, low-fat yogurt, fruit yogurt, hard cheese, soft cheese, ice cream	Reverse	Reverse	Reverse

Positive indicates that higher consumption of this food group received higher scores. Reverse indicates that higher consumption of this food group received lower scores. In the general PVG food pattern, consumption of whole grains and refined grains was aggregated as the "grains" food group.

Supplementary Table S2. Associations of pro-vegetarian (PVG) Food Patterns with nutritional factors in cancer-free participants from the Moli-sani Study cohort (n=22,081)

	Change in nutritional factor								
	General PVG			Healthful PVG			Unhealthful PVG		
Nutritional factors	Regression coefficient β	SE	p-value	Regression coefficient β	SE	p-value	Regression coefficient β	SE	p-value
Vegetables	6.18	0.07	<.0001	6.42	0.07	<.0001	-5.40	0.06	<.0001
Fruits	10.49	0.25	<.0001	13.39	0.22	<.0001	-9.53	0.21	<.0001
Legumes	0.75	0.01	<.0001	0.81	0.01	<.0001	-0.69	0.01	<.0001
Whole grains	0.27	0.03	<.0001	0.53	0.02	<.0001	-0.43	0.02	<.0001
Refined grains	1.89	0.09	<.0001	-2.17	0.08	<.0001	4.63	0.07	<.0001
Cereals	2.32	0.08	<.0001	-1.12	0.07	<.0001	4.06	0.06	<.0001
Potatoes	0.84	0.02	<.0001	-0.57	0.02	<.0001	0.12	0.02	<.0001
Nuts	0.06	0.00	<.0001	0.06	0.00	<.0001	-0.05	0.00	<.0001
Olive oil	0.71	0.01	<.0001	0.63	0.01	<.0001	-0.60	0.01	<.0001
Coffee/tea	-0.42	0.09	<.0001	2.32	0.08	<.0001	-2.44	0.07	<.0001
Fruit juice	-0.07	0.07	0.28	-0.23	0.06	0.0002	0.10	0.06	0.08
Sugary beverages	-0.47	0.08	<.0001	-0.67	0.07	<.0001	0.82	0.06	<.0001
Sweets	-0.77	0.05	<.0001	-1.00	0.04	<.0001	1.61	0.04	<.0001
Meat and meat products	-2.54	0.04	<.0001	-1.96	0.04	<.0001	-1.46	0.04	<.0001
Animal fat	-0.11	0.00	<.0001	-0.08	0.00	<.0001	-0.06	0.00	<.0001
Eggs	-0.44	0.01	<.0001	-0.37	0.01	<.0001	-0.35	0.01	<.0001
Fish	-0.55	0.03	<.0001	-0.24	0.03	<.0001	-1.39	0.03	<.0001
Dairy products	-4.91	0.13	<.0001	-1.52	0.13	<.0001	-3.82	0.11	<.0001
Energy intake (kcal/d)	-8.78	0.63	<.0001	7.93	0.59	<.0001	-3.47	0.54	<.0001
Fibre	0.47	0.005	<.0001	0.45	0.005	<.0001	-0.26	0.005	<.0001
Alcohol (g/d)	-0.32	0.02	<.0001	0.11	0.02	<.0001	0.21	0.02	<.0001
Carbohydrate (% TEI)	0.37	0.01	<.0001	0.04	0.01	<.0001	0.43	0.01	<.0001
Protein (% TEI)	-0.14	0.002	<.0001	-0.10	0.002	<.0001	-0.11	0.002	<.0001
Fat (% TEI)	-0.11	0.01	<.0001	0.02	0.01	<.0001	-0.36	0.005	<.0001

SE=standard error; TEI=total energy intake. Change for a 1-unit increase in three food patterns. P values were obtained from linear regression models adjusted for sex, age, and energy intake.

Supplementary Title for Figure S2. Association between adherence to a) healthful pro-vegetarian food pattern; and b) unhealthful pro-vegetarian food pattern with cancer risk in participants from the Moli-sani study cohort (n = 22,081).

Legend for Figure S2. Cancer-free survival curves were obtained from the multivariable model adjusted for age, sex, energy intake, alcohol intake, residence, educational level, housing tenure, occupational class, smoking status, body mass index, leisure-time physical activity, history of CVD, diabetes, hypertension, hyperlipidaemia, aspirin use, oral contraception use, hormone replacement therapy, menopausal status, and family history of cancer. Cancer-free survival curves were generated using the first imputed dataset. The other imputed datasets were similar and thus omitted.

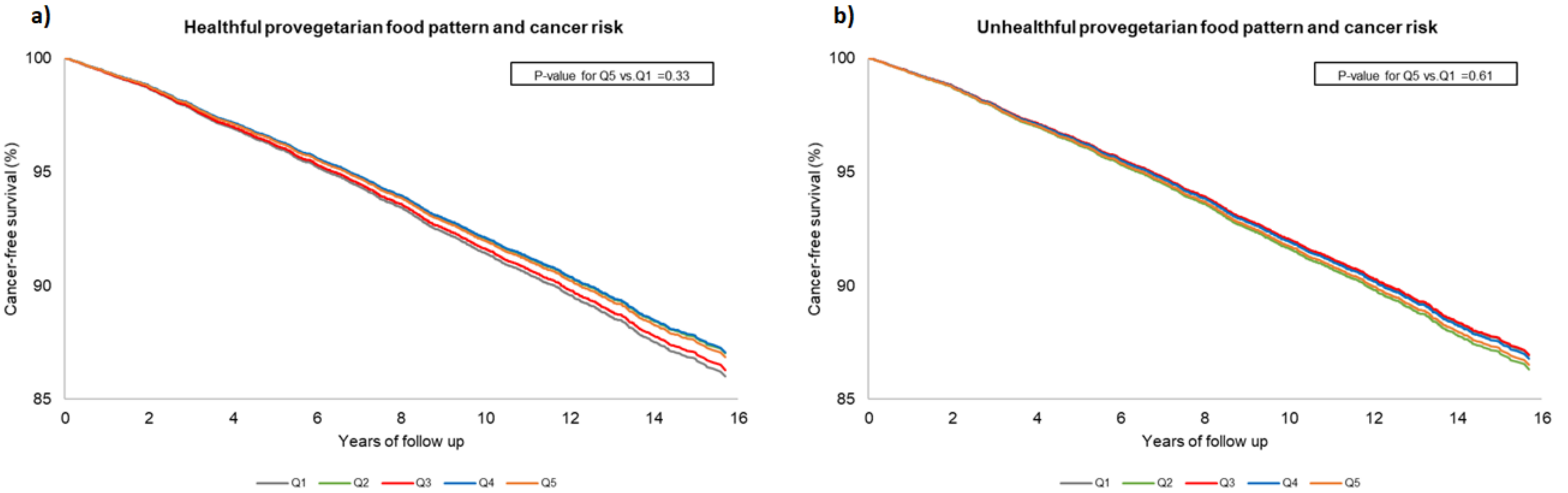

Supplementary Appendix

The enrolment phase of the Moli-sani Study was conducted at the Research Laboratories of the Catholic University in Campobasso (Italy), the follow up of the Moli-sani cohort is being conducted at the Department of Epidemiology and Prevention of the IRCCS Neuromed, Pozzilli, Italy.

Steering Committee: Licia Iacoviello*°(Chairperson), Giovanni de Gaetano* and Maria Benedetta Donati*.
Scientific Secretariat: Chiara Cerletti*, Marialaura Bonaccio*, Americo Bonanni*, Simona Costanzo*, Amalia De Curtis*, Augusto Di Castelnuovo§, Alessandro Gialluisi*°, Francesco Gianfagna°§, Mariarosaria Persichillo*, Teresa Di Prospero* (Secretary).
Safety and Ethical Committee: Jos Vermylen (Catholic University, Leuven, Belgio) (Chairperson), Renzo Pegoraro (Pontificia Accademia per la Vita, Roma, Italy), Antonio Spagnolo (Catholic University, Roma, Italy).
External Event Adjudicating Committee: Deodato Assanelli (Brescia, Italy), Livia Rago (Campobasso, Italy).
Baseline and Follow-up Data Management: Simona Costanzo* (Coordinator), Marco Olivieri (Campobasso, Italy), Teresa Panzera*.
Data Analysis: Augusto Di Castelnuovo§ (Coordinator), Marialaura Bonaccio*, Simona Costanzo*, Simona Esposito*, Alessandro Gialluisi*°, Francesco Gianfagna°§, Sabatino Orlandi*, Emilia Ruggiero*, Alfonsina Tirozzi*.
Biobank, Molecular and Genetic Laboratory: Amalia De Curtis* (Coordinator), Sara Magnacca§, Fabrizia Noro*, Alfonsina Tirozzi*.
Recruitment Staff: Mariarosaria Persichillo* (Coordinator), Francesca Bracone*, Teresa Panzera*.
Communication and Press Office: Americo Bonanni*.
Regional Institutions: Direzione Generale per la Salute – Regione Molise; Azienda Sanitaria Regionale del Molise (ASReM, Italy); Agenzia Regionale per la Protezione Ambientale del Molise (ARPA Molise, Italy); Molise Dati Spa (Campobasso, Italy); Offices of vital statistics of the Molise region.
Hospitals: Presidi Ospedalieri ASReM: Ospedale A. Cardarelli – Campobasso, Ospedale F. Veneziale – Isernia, Ospedale San Timoteo – Termoli (CB), Ospedale Ss. Rosario – Venafro (IS), Ospedale Vietri – Larino (CB), Ospedale San Francesco Caracciolo – Agnone (IS); Casa di Cura Villa Maria – Campobasso; Ospedale Gemelli Molise – Campobasso; IRCCS Neuromed – Pozzilli (IS).

*Department of Epidemiology and Prevention, IRCCS Neuromed, Pozzilli, Italy
°Department of Medicine and Surgery, University of Insubria, Varese, Italy
§Mediterranea Cardiocentro, Napoli, Italy

Moli-sani Study Past Investigators are available at https://www.moli-sani.org/?page_id=173
